# Interleukin-15 deficient rats have reduced osteopontin at the maternal-fetal interface

**DOI:** 10.3389/fcell.2023.1079164

**Published:** 2023-04-20

**Authors:** Kelly J. Baines, Michelle S. Klausner, Violet S. Patterson, Stephen J. Renaud

**Affiliations:** ^1^ Department of Anatomy and Cell Biology, Schulich School of Medicine and Dentistry, University of Western Ontario, London, ON, Canada; ^2^ Children’s Health Research Institute, Lawson Health Research Institute, London, ON, Canada

**Keywords:** natural killer cells, decidua, osteopontin, placenta, trophoblast, interleukin-15

## Abstract

**Introduction:** Uterine Natural Killer (NK) cells are the predominant immune cells within the decidua during early pregnancy. These cells are thought to regulate aspects of decidualization and placental development, but their functions remain poorly characterized, especially in species with deeply invading trophoblasts such as humans and rats. Interleukin-15 (IL-15) is a cytokine required for NK cell development and survival. IL-15 mutant (*IL15Δ/Δ*) rats lack NK cells and exhibit altered placental development with precocious trophoblast invasion. In this study, we profiled gene expression differences between wild-type and *IL15Δ/Δ* implantation sites to reveal candidate factors produced by uterine NK cells that may regulate placentation and trophoblast invasion.

**Methods:** Clariom S gene expression profiling was performed using implantation sites collected from pregnant wild-type and *IL15Δ/Δ* rats on gestational day 9.5. Levels and localization of perforin and osteopontin in implantation sites from wild-type and *IL15Δ/Δ* rats were further analyzed. The effect of osteopontin on the invasive capacity of rat trophoblasts was evaluated using Matrigel-based Transwell assays.

**Results:** There were 257 genes differentially expressed between wild-type and *IL15Δ/Δ* implantation sites on gestational day 9.5, including decreased expression of various NK cell markers in *IL15Δ/Δ* rats, as well as *Spp1*, which encodes osteopontin. In wild-type rats, osteopontin was present within the decidua basalis and adjacent to the primitive placenta, and osteopontin colocalized with the NK cell marker perforin. Osteopontin was also detectable in uterine glands. Conversely, in *IL15Δ/Δ* rats, osteopontin and perforin were not readily detectable in the decidua despite robust osteopontin levels in uterine glands. Neutralization of osteopontin in media conditioned by cells isolated from the decidua decreased invasion of rat trophoblasts, suggesting that reduced levels of osteopontin are unlikely to account for the precocious trophoblast invasion in *IL15Δ/Δ* rats.

**Conclusion:** Osteopontin is expressed by NK cells at the maternal-fetal interface in rats and may contribute to modulation of trophoblast invasion.

## 1 Introduction

Decidualization involves morphological and functional changes to the uterine endometrium to support embryo implantation, and is a vital process for humans, rodents, and other species possessing hemochorial placentation. Decidualization is characterized by transformation of endometrial stromal cells into epithelioid decidual stromal cells, maturation of uterine glands, and a robust accumulation of immune cells. In particular, uterine natural killer (NK) cells are the predominant immune cells that populate the decidua in both humans and rodents during early pregnancy, comprising over 70% of uterine leukocytes (Jabrane-Ferrat, 2019). Uterine NK cells are found in close proximity to spiral arteries (the feeder vessels of the placenta) and are thought to be important for placental and decidual development (Cerdeira et al., 2013). Studies using mouse models strongly suggest that NK cells are active participants in remodeling spiral arteries to promote increased blood flow to the placenta prior to rapid fetal growth in late gestation (Charalambous et al., 2012; Moffett and Colucci, 2014; Filipovic et al., 2018). The role of uterine NK cells at the maternal-fetal interface in other species, such as rats and humans, is less clear since deeply-invading trophoblasts emanating from the placenta are chiefly responsible for spiral artery remodeling in these species (Soares et al., 2012). It has been proposed that uterine NK cells prepare spiral arteries for the arrival of invading trophoblasts, or help control the extent of trophoblast-directed remodeling, but the mechanisms are poorly understood (Pijnenborg et al., 1981; Lash et al., 2010; Wallace et al., 2012; Gaynor and Colucci, 2017). Since dysregulated trophoblast invasion is highly associated with serious pregnancy complications in humans including early-onset preeclampsia and fetal growth restriction (hypo-invasion) and placenta accreta spectrum (hyper-invasion), deciphering how NK cells modulate the depth and extent of trophoblast invasion is of critical importance.

Interleukin-15 (IL-15) is a cytokine required for NK cell development and homeostasis. In the absence of IL-15, the number of NK cells (as well as other innate lymphoid cells including invariant NKT cells and TCRγδ T cells) is drastically reduced (Huntington et al., 2007; Gillgrass et al., 2014; DeGottardi et al., 2016). IL-15 interacts with a heterotrimeric receptor complex composed of a unique IL-15 receptor *α* subunit that binds with high affinity to IL-15, as well as a *β* subunit and common *γ* chain shared with the IL-2 receptor. To examine the role of uterine NK cells in the regulation of decidualization and placentation, several studies using mice have disrupted the gene encoding IL-15 or its receptor to generate NK cell-deficient animals. These studies describe uterine and placental abnormalities that result from an absence of uterine NK cells, including a hypocellular decidua and failed spiral artery remodeling (Ashkar et al., 2003; Barber and Pollard, 2003; Mori et al., 2016). An IL-15 mutant (*IL15Δ/Δ*) rat model has also been generated, which both complements and adds to findings from studies using IL-15 deficient mice (Renaud et al., 2017). At mid-gestation, *IL15Δ/Δ* rats have an expanded junctional zone (the portion of the rodent placenta where invasive trophoblasts arise) and dilated spiral arteries with increased internal luminal diameters compared to wild-type (WT) rats due to precocious trophoblast invasion (Renaud et al., 2017). The enhanced trophoblast invasion in *IL15Δ/Δ* rats is consistent with results using a transient NK cell depletion strategy involving systemic injection of asialo GM1 antibodies (Chakraborty et al., 2011), suggesting that the cause of the precocious trophoblast invasion in these models is due to reduced numbers of NK cells.

Since *IL15Δ/Δ* rats lack NK cells and exhibit hyper-invasive trophoblasts, we hypothesized that uterine NK cells produce factors that contribute to the regulation of trophoblast invasion. Herein, we performed gene expression profiling of rat WT and *IL15Δ/Δ* implantation sites on GD 9.5, a time when uterine NK cells are prevalent in the decidua and placentation is in its early stages, to deduce putative factors produced by uterine NK cells. We identified a variety of differentially expressed genes between WT and *IL15Δ/Δ* dams, most notably *Spp1*, the gene encoding the multifunctional phosphoprotein osteopontin (OPN). These findings advance our understanding of the role of IL-15 in regulating decidual immune cell function and placentation, and may potentially provide insight into mechanisms of placenta-related pregnancy complications associated with dysregulated trophoblast invasion.

## 2 Materials and methods

### 2.1 Animals

WT male (8–10 weeks old) Holtzman Sprague-Dawley rats were obtained from Envigo and bred with in-house generated WT or *IL15Δ/Δ* Holtzman Sprague-Dawley females (6–8 weeks old). Experiments involving isolation of decidual cells were conducted with WT Sprague-Dawley animals (Charles River). All animals were maintained in a 12 h light: 12 h dark cycle with water and food available *ad libitum*. Females were cycled by daily inspection of cells within the vaginal lavage and mated with a fertile male when in proestrus. Cells were identified under a microscope as outlined in Goldman et al. (2007). Gestational day (GD) 0.5 was defined as the day immediately following mating if spermatozoa were detected within the vaginal lavage. All protocols involving the use of rats were approved by the University of Western Ontario Animal Care and Use Committee, and procedures conducted in accordance with the Canadian Council on Animal Care.

### 2.2 Tissue collection

Pregnant dams were sacrificed on GD 9.5 using mild carbon dioxide inhalation until respiratory failure, followed by thoracotomy. Whole GD 9.5 implantation sites (referring to sites in the uterus containing decidualized endometrium along with nascent embryonic and extraembryonic structures) were collected and placed in sterile saline for cell isolation experiments. For immunohistochemistry, whole implantation sites were immersed in 10% neutral buffered formalin and then embedded in paraffin. For all other experiments, implantation sites were snap frozen in liquid nitrogen and stored at −80°C prior to analyses.

### 2.3 Clariom S gene expression array

RNA was isolated from 3 snap frozen GD 9.5 implantation sites randomly chosen from each of 3 WT and 3 *IL15Δ/Δ* rats at GD 9.5 (a total of 9 implantation sites per group). RNA was extracted by homogenizing whole implantation sites in RiboZol (VWR), followed by collection of the aqueous phase which was applied to RNeasy columns (Qiagen). Following DNase I (Qiagen) treatment, purified RNA (30 µg per sample) from the 3 implantation sites per dam were combined to generate 3 samples per group, and samples were submitted to Hamilton Health Sciences Centre (Hamilton, ON, Canada) for transcriptome analysis on a GeneChip Scanner 3000 using the Clariom S rat array (ThermoFisher Scientific), as previously described (Roberts et al., 2021). Samples were normalized using the SST-RMA (Signal Space Transformation- Robust Multiarray Analysis) data normalization algorithm to reduce background. Data files were generated and processed for analysis using Transcriptome Analysis Console Software 4.0 (ThermoFisher Scientific) to analyze global gene expression patterns. Gene ontology pathway analysis was completed using DAVID Functional Annotation Bioinformatics (Huang et al., 2009).

### 2.4 Quantitative RT-PCR

RNA was extracted from cells and tissues by lysing in RiboZol as directed by the manufacturer. Purified RNA was used to make cDNA using High-Capacity cDNA Reverse Transcription kit (ThermoFisher Scientific), diluted 1:10, and used for quantitative RT-PCR (qRT-PCR). Diluted cDNA was mixed with SensiFAST SYBR green PCR Master Mix (FroggaBio) and primers detailed in Table 1. To amplify DNA and detect fluorescence, a CFX Connect Real-Time PCR system (Bio-Rad Laboratories) was used. Cycling conditions involved an initial holding step (95°C for 3 min), followed by 40 cycles of two-step PCR (95°C for 10 s, 60°C for 45 s), and a dissociation step (65°C for 5 s, and a sequential increase to 95°C). To ensure decidual samples were not contaminated by uterine glands, samples with high expression (>5-fold increase compared to the mean of all samples) of the glandular marker *Foxa2* were excluded from further qRT-PCR analysis. The comparative cycle threshold (ΔΔCt) method was used to calculate relative mRNA expression, using the geometric mean of Ct values obtained from amplification of four genes (*Rn18s*, *Ywhaz*, *Actb*, and *Gapdh*) as reference RNA.

**TABLE 1:** Forward and reverse primers used for quantitative RT-PCR amplification.

Gene	Accession number	Forward sequence	Reverse sequence
*Rn18s*	NM_046237.1	5′-GCA​ATT​ATT​CCC​CAT​GAA​CG-3′	5′-GGC​CTC​ACT​AAA​CCA​TCC​AA-3′
*Actb*	NM_031144.3	5′-GCC​ATG​TAC​GTA​GCC​ATC​C-3′	5′-CTC​TCA​GCT​GTG​GTG​GTG​AA-3′
*Bmp4*	NM_012827.2	5′-GAA​GAA​CAT​CTG​GAG​AAC​ATC-3′	5′-GGG​CTT​CAT​AAC​CTC​ATA​AAT-3′
*Ccl5*	NM_031116.3	5′-CCT​TGC​AGT​CGT​CTT​TGT​CA-3′	5′- GAG​TAG​GGG​GTT​GCT​CAG​TG-3′
*Crym*	NM_053955.2	5′-GCT​GTT​GGA​GCC​AGT​AGA​CC-3′	5′-TCA​GCC​CCT​GAC​AAC​AGA​AC-3′
*Eomes*	XM_039082697.1	5′-TTC​ACC​CAG​AAT​CTC​CCA​AC-3′	5′-TGG​AAG​GCT​CAT​TCA​AGT​CC-3′
*Esrrb*	NM_001008516.3	5′- TGC​CTG​AAG​GGG​ATA​TCA​AG-3′	5′- TGC​CAG​CTT​GTC​ATC​ATA​GG-3′
*Foxa2*	NM_001399085.1	5′-ACG​GTG​CCA​TAG​CTG​ACT​TT-3′	5′- CAC​GGA​AGA​GTA​GCC​CTC​AG-3′
*Gapdh*	NM_017008.4	5′-AGA​CAG​CCG​CAT​CTT​CTT​GT-3′	5′-CTT​GCC​GTG​GGT​AGA​GTC​AT-3′
*Id2*	NM_013060.4	5′-TGA​AAG​CCT​TCA​GTC​CGG​TG-3′	5′-GAG​CTT​GGA​GTA​GCA​GTC​GT-3′
*Itgav*	NM_001398693.1	5′-TTC​CCT​GAA​GTC​ATC​CGC​TT-3′	5′-GAA​CCG​CCA​AGA​TGA​TCA​CC-3′
*Itga5*	NM_001108118.1	5′-AGG​TGA​CGG​GAC​TCA​ACA​AC-3′	5′-GGG​CAT​TTC​AGG​ACT​TGT​GT-3′
*Itgb1*	NM_017022.2	5′-GCC​AGT​GTC​ACC​TGG​AAA​AT-3′	5′-TGT​GCC​CAC​TGC​TGA​CTT​AG-3′
*Itgb3*	NM_153720.2	5′-TGA​CAT​CGA​GCT​GGT​GAA​AG-3′	5′-GTA​GCA​AGG​CCA​ATG​AGC-3′
*Prf1*	NM_017330.2	5′-GGC​ACT​CAA​GGA​ACC​TTC​C-3′	5′-CTC​AAG​CAG​TCT​CCT​ACC-3′
*Spp1*	NM_012881.2	5′-AGA​CTG​GCA​GTG​GTT​TGC​TT-3′	5′-TGT​AAT​GCG​CCT​TCT​CCT​CT-3′
*Tpbpa*	NM_172073.1	5′- TGG​AGA​GCG​GAG​ATG​AGA​TT-3′	5′- GGG​ACT​GGC​TAC​TGA​GTT​GG-3′
*Vim*	NM_031140.1	5′-ATG​CTT​CTC​TGG​CAC​GTC​TT-3′	5′-TGG​CAG​CCA​CAC​TTT​CAT​AC-3′
*Ywhaz*	NM_013011.3	5′-TTG​AGC​AGA​AGA​CGG​AAG​GT-3′	5′-CCT​CAG​CCA​AGT​AGC​GGT​AG-3′

### 2.5 Protein extraction and Western blotting

Total tissue protein was isolated by homogenizing snap frozen GD 9.5 implantation sites in radioimmunoprecipitation assay lysis buffer (50 mM Tris, 150 mM NaCl, 1% Nonidet P-40, 0.5% sodium deoxycholate, 0.1% SDS, pH 7.2) supplemented with protease inhibitor cocktail (Sigma-Aldrich). Homogenates were then sonicated (Sonic Dismembrator Model 100, ThermoFisher Scientific). To measure total protein concentrations, a modified bicinchoninic acid assay (Bio-Rad Laboratories) was used. Approximately 25 µg of tissue lysates were mixed with 4× reducing loading buffer (0.25 M Tris, 8% SDS, 30% glycerol, 0.02% bromophenol blue, 0.3 M dithiothreitol). Samples were boiled for 5 min and then subjected to SDS-polyacrylamide gel electrophoresis. Proteins were transferred to a nitrocellulose membrane, blocked with 3% bovine serum albumin (BSA) in TBS that contained 0.1% Tween-20, and then probed with primary antibodies specific for Perforin (PRF; TP251, 1:1000, Torrey Pines Biolabs) and Glyceraldehyde 3-phosphate dehydrogenase (GAPDH; 5174, 1:1000, Cell Signaling Technology). Following incubation with species-appropriate, fluorescent dye-conjugated secondary antibodies (Cell Signaling Technology), protein signals were detected using a LI-COR Odyssey imaging system (LI-COR Biotechnology). Band intensities of target proteins were normalized to corresponding GAPDH signals using ImageJ software (Schneider et al., 2012).

### 2.6 Immunohistochemistry

Whole implantation sites on GD 9.5 were collected, fixed in 10% neutral buffered formalin, processed, paraffin-embedded, and sectioned at 5 µm thickness. Serial sections were deparaffinized in Histoclear (National Diagnostics) and rehydrated using increasing dilutions of ethanol washes. Formaldehyde crosslinks were fragmented by placing slides in Reveal Decloaker (Biocare Medical) at 95°C for 20 min. Following rehydration, endogenous peroxidases were blocked by treating tissues with 0.3% hydrogen peroxide in methanol. Sections were then permeabilized using 0.3% Triton-X and 1% BSA in PBS and blocked with 10% normal goat serum (ThermoFisher Scientific). Sections were immersed in primary antibodies specific for OPN (0.5 μg/mL, sc-21742, Santa Cruz Biotechnology) or PRF (2.5 μg/mL, TP251, Torrey Pines Biolabs) overnight at 4°C. Immersion of sections in 2.5 μg/mL rabbit IgG (in place of PRF) or 0.5 μg/mL mouse IgG_1_ (in place of OPN) were used as negative controls to ensure antibody specificity. Subsequently, for chromogenic staining, sections were incubated with species-appropriate biotinylated secondary antibodies, followed by Vectastain (Vector Laboratories). Color was developed using 3-amino-9-ethylcarbazole red (Vector Laboratories), counterstained with hematoxylin (Sigma-Aldrich) and mounted with Fluoromount-G mounting medium (Southern Biotech). For fluorescent staining, sections were incubated with species-specific Alexa 488-conjugated or Alexa 555-conjugated secondary antibodies followed by 4′,6-diamidino-2-phenylindole (DAPI) nuclear stain (ThermoFisher Scientific). Sections were imaged using a Nikon ECLIPSE Ni series microscope equipped with a Ds-Qi2 camera.

### 2.7 Isolation and culture of cells from the decidua

Whole implantation sites on GD 9.5 were collected in sterile saline and placed under a dissection microscope. After removing the nascent embryo and placenta, decidual tissue was isolated, minced, and then digested in Gentle Collagenase/Hyaluronidase in DMEM (07919, StemCell Technologies) for 2 h at 37°C with moderate agitation as per the manufacturer’s instructions. Digested tissue was passed through a 100 μm cell strainer, and the resulting single cell suspension was layered over Lymphoprep (StemCell Technologies). After centrifugation, the buffy coat containing platelets, leukocytes, and other mononuclear cells was carefully aspirated, washed, and resuspended in RPMI-1640 medium supplemented with 1% fetal bovine serum (FBS) and 10 ng/mL rat recombinant IL-15 (PeproTech). Cells were then placed in cell culture plates at 1.0 × 10^6^ cells/mL in a gas and temperature-controlled incubator (5% CO_2_, 37°C) for 24 h. After the incubation, conditioned media containing suspended cells were removed and centrifuged at 300 × g for 10 min. The supernatant was aliquoted and stored at −20°C as a source of decidual conditioned media and to measure levels of OPN. RNA was isolated from both the pelleted cells and the cells that adhered to the cell culture plate to evaluate expression of NK cell markers and *Spp1*.

### 2.8 Immunofluorescence

After resuspension, isolated decidual cells in suspension were fixed for 5 min in 4% paraformaldehyde, and then smeared and heat-fixed on gelatin-coated microscope slides. Cell smears were permeabilized using 0.3% Triton-X and 1% BSA in PBS, blocked with 10% normal goat serum (ThermoFisher Scientific), and immersed in primary antibodies specific for OPN (0.5 μg/mL, sc-21742, Santa Cruz Biotechnology) and PRF (2.5 μg/mL, TP251, Torrey Pines Biolabs) overnight. Slides were then incubated with Alexa 555-conjugated anti-rabbit secondary (ThermoFisher Scientific) and Alexa 488-conjugated anti-mouse secondary (ThermoFisher Scientific) antibodies for 1 h at room temperature. Nuclei were then stained with DAPI (ThermoFisher Scientific), and slides mounted with Fluoromount-G. Images were acquired using a Nikon ECLIPSE Ni Series microscope equipped with a Ds-Qi2 camera.

### 2.9 Enzyme-linked immunosorbent assay (ELISA)

The levels of OPN secreted into medium conditioned for 24 h by isolated decidual cells were determined by a Mouse/Rat OPN Quantikine ELISA kit (MOST00, R&D systems). The procedure was performed using a protocol provided by the manufacturer. The sensitivity of detection of this assay is 8.5 pg/mL.

### 2.10 Rat TS cell culture

Blastocyst-derived rat trophoblast stem (TS) cells were used to evaluate the effects of OPN on trophoblast cell invasion. Rat TS cells were generously provided by Michael Soares (University of Kansas Medical Center, Kansas City, KS), and were cultured as described previously (Asanoma et al., 2011). Briefly, TS cells were maintained in RPMI-1640 medium (ThermoFisher Scientific) supplemented with 20% (v/v) FBS (ThermoFisher Scientific), 100 μM 2-mercaptoethanol (Sigma-Aldrich), 1 mM sodium pyruvate (Sigma-Aldrich), 50 units/mL penicillin, 50 μg/mL streptomycin, fibroblast growth factor 4 (25 ng/mL; R&D Systems), activin A (10 ng/mL, R&D systems) and heparin (1 μg/mL; Sigma-Aldrich). 70% of the media was preconditioned by mitomycin C–treated mouse embryonic fibroblasts prior to being added to rat TS cells. Cells were maintained at 37°C with 5% CO_2_, and were subcultured by light trypsinization prior to reaching confluency. To induce differentiation, TS cells were cultured for 6 days as above but without mouse embryonic fibroblast-conditioned media, fibroblast growth factor 4, activin A, or heparin. Media were replenished daily.

### 2.11 Matrigel-based invasion assay

Transwells (6.5 mm, 8 µm pore, Greiner BioOne) were coated with growth factor-reduced Matrigel (BD Biosciences, 400 μg/mL diluted in serum free RPMI-1640 medium) for 3 h. Medium was removed prior to plating cells. Rat TS cells were differentiated for 6 days, and then approximately 1.0 × 10^4^ cells were placed in the Transwells on top of the Matrigel. Transwells were then positioned in wells containing decidual conditioned medium supplemented with either PBS, normal goat IgG (1 μg/mL, AB-108-C, R&D Systems), or an OPN neutralizing antibody (1 μg/mL, AF808, R&D Systems), and incubated for 24 h at 37°C, 5% CO_2_. After 24 h, excess cells and Matrigel were discarded from the top of the chamber using a cotton swab, and cells that invaded through to the underside of the Transwell were fixed in methanol and stained using Diff-Quik (GE Healthcare). Membranes were removed from the Transwell, placed on slides, and invaded cells counted under a microscope. For each condition, the total number of cells that invaded in 3 random fields of view per membrane was counted, and three independent membranes were used per experiment. Counts were averaged and normalized to the number of cells that invaded in the control condition to facilitate comparisons between experiments.

### 2.12 Statistical analysis

Clariom S analysis was described previously. When analyzing gene expression data in which multiple implantation sites were collected from each dam, statistical significance was determined by a linear mixed model using the lme4, lmerTest, and emmeans packages in R to control for potential litter effects, followed by a Tukey’s *post hoc* test for multiple comparisons (Bates et al., 2014; Kuznetsova et al., 2017; Lenth, 2023). For all other experiments, statistical significance was determined by Student’s *t*-test when comparing two groups, and analysis of variance followed by a Tukey’s *post hoc* test when comparing three groups. Differences were considered statistically significant when *p* < 0.05. GraphPad Prism 8.0 was used for all graphing and statistical analysis. All animal experiments were conducted using a minimum of 3 dams. The number of animals and statistical analysis used in each experiment are specified in figure legends.

## 3 Results

### 3.1 Gene expression profiling of implantation sites from WT and IL15Δ/Δ rats

**FIGURE 1:**
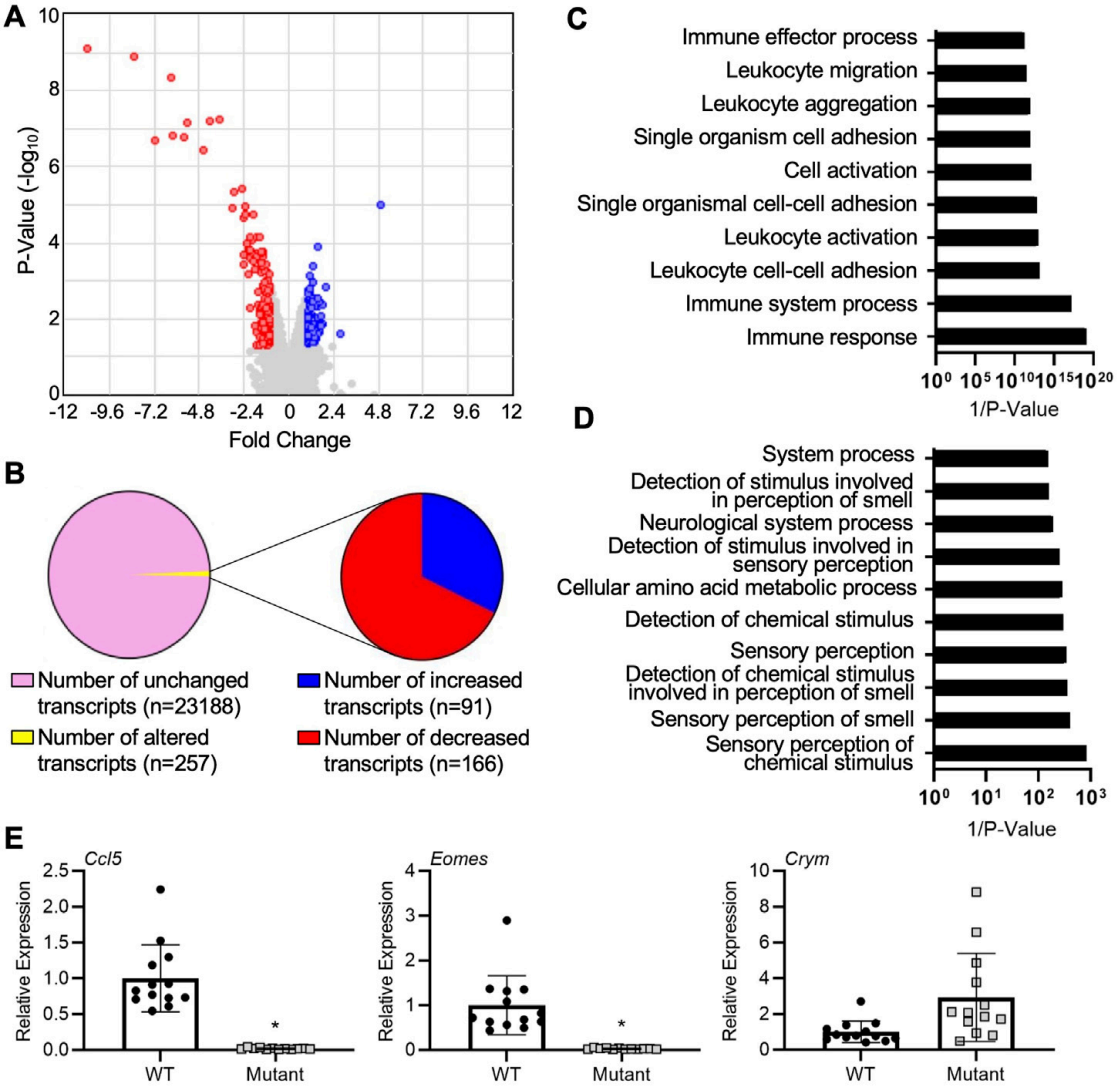
Gene expression profiling of implantation sites from WT and *IL15Δ/Δ* rats. **(A)** Volcano plot showing the number of unique transcripts identified in the Clariom S analysis. Transcripts significantly (fold change >2, *p* < 0.05) upregulated in implantation sites from *IL15Δ/Δ* rats compared to WT rats are shown in blue, and downregulated transcripts are shown in red. The *x*-axis represents magnitude of fold change, and the *y*-axis shows *p*-value. **(B)** Pie chart showing the number of transcripts upregulated and downregulated (2-fold, *p* < 0.05) in implantation sites from *IL15Δ/Δ* rats compared to WT rats. **(C)** Top 10 gene ontology pathways downregulated in implantation sites from *IL15Δ/Δ* rats. **(D)** Top 10 gene ontology pathways upregulated in implantation sites from *IL15Δ/Δ* rats. Pathway analysis was conducted by inputting transcripts changed more than 2-fold with a *p* < 0.05 in *IL15Δ/Δ* rats compared to WT rats into DAVID Bioinformatics Resource (*N* = 3 for each group). **(E)** Quantitative RT-PCR was used to compare the relative expression of select transcripts in a separate cohort of GD 9.5 implantation sites from *IL15Δ/Δ* rats (mutant) and WT rats. *N* ≥ 13 implantation sites from at least 5 dams per group. Results represent means ± SEM, and were analyzed using a linear mixed model followed by Tukey’s *post hoc* test. Data significantly different from controls (*p* < 0.05) are indicated by an asterisk (*).

We first sought to uncover global gene expression differences between WT and *IL15Δ/Δ* rats in order to determine candidate genes that may underlie the robust changes in decidualization and placentation observed in *IL15Δ/Δ* rats. Overall, from a total of 23,418 transcripts examined, there were significant (>2-fold altered expression, *p* < 0.05) differences in the expression of 257 genes between *IL15Δ/Δ* and WT rat implantation sites (Figures 1A, B). Of these, 91 transcripts were upregulated in *IL15Δ/Δ* rats, and 166 transcripts were downregulated (Figure 1B). The top 20 upregulated and downregulated genes in *IL15Δ/Δ* rat implantation sites are shown in Tables 2, 3, respectively. Among transcripts upregulated in *IL15Δ/Δ* rats, genes associated with neurotransmission and central nervous system development were prominent (e.g., *Gria4*, 3.9-fold; *Crym*, 3.6-fold) as well as various glycoproteins involved in implantation (e.g., *Zp2*, 31.2-fold; *Hp*, 6.8-fold). Many of the genes exhibiting decreased expression in *IL15Δ/Δ* rats encoded proteins associated with inflammatory processes and immune cell development (e.g., *Gzmc*, 1798.9-fold; *Prf1*, 318-fold; *Gzmb*, 80.4-fold), which was expected given the diminished presence of uterine NK cells in *IL15Δ/Δ* rats.

**TABLE 2:** List of top 20 upregulated genes in IL15Δ/Δ rat conceptuses.

Gene	Fold change	WT reads (Log_2_)	*IL15Δ/Δ* reads (Log_2_)
*Zp2*	31.18	3.27	8.23
*Hp*	6.84	8.42	11.19
*Gria4*	3.92	5.23	7.2
*LOC102553649*	3.61	7.87	9.72
*Crym*	3.61	5.13	6.98
*Olr1220*	3.36	4.34	6.09
*Olr1468*	3.29	2.92	4.63
*Chpt1*	3.17	12.26	13.93
*Olr1081*	3.09	3.15	4.78
*Olr84*	2.98	2.32	3.9
*LOC685828*	2.93	6.98	8.53
*LOC100911939*	2.93	3.33	4.88
*Olr1237*	2.93	3.33	4.88
*Olr1237*	2.93	3.33	4.88
*Fis1*	2.91	8.62	10.16
*Samt4*	2.87	2.84	4.36
*Hormad1*	2.82	4.25	5.75
*Qpct*	2.72	7.83	9.27
*Vom2r10*	2.72	3.36	4.81
*Olr1063*	2.67	4.55	5.97

**TABLE 3:** List of top 20 downregulated genes in IL15Δ/Δ rat conceptuses.

Gene	Fold change	WT reads (Log_2_)	*IL15Δ/Δ* reads (Log_2_)
*Gzmc*	−1798.9	15.71	4.9
*Prf1*	−318	13.13	4.82
*LOC100911163*	−140.18	11.64	4.51
*Gzmb*	−80.41	11.12	4.79
*Gzmbl2*	−75.92	11.32	5.08
*Nkg7*	−49.08	8.74	3.13
*Il2rb*	−42.81	9.34	3.92
*Spp1*	−23.94	12.21	7.63
*Fcrl6*	−18.35	8.44	4.24
*Cd96*	−13.34	7.42	3.68
*Ccl5*	−7.97	6.93	3.94
*Eomes*	−7.86	8.27	5.29
*Laptm5*	−5.57	9.17	6.69
*Mcpt10*	−5.39	8.07	5.64
*LOC100910060*	−5.36	5.54	3.12
*Plek*	−5.19	5.42	3.04
*Lcp1*	−5.17	10.04	7.67
*Clnk*	−5.16	6.0	3.63
*Mcpt8*	−4.86	8.35	6.06
*Sh2d1b*	−4.49	5.56	3.4

The top gene ontology pathway terms for genes downregulated in *IL15Δ/Δ* implantation sites included immune response (*p* = 4.2E-6, 12 genes), immune system process (*p* = 3.3E-4, 10 genes), and leukocyte cell-cell adhesion (*p* = 3.0E-5, 5 genes, Figure 1C). Interestingly, pathways associated with upregulated genes were related to neurological processes and sensory perceptions including sensory perception of chemical stimulus (*p* = 1.2E-3, 13 genes) and sensory perception of smell (*p* = 2.5E-3, 12 genes, Figure 1D). To validate a subset of results in a separate cohort of implantation sites from WT and *IL15Δ/Δ* dams, qRT-PCR was conducted. The relative expression of *Crym*, which encodes the thyroid hormone binding protein μ-crystallin, appeared to show a similar ∼3-fold increase in expression in *IL15Δ/Δ* rat implantation sites as was detected using Clariom S analysis, although levels were variable and it did not reach statistical significance (*p* = 0.17). Consistent with the Clariom S analysis, the relative expression of genes encoding eomesodermin (*Eomes*), a T-box transcription factor required for NK cell development, and chemokine ligand 5 (*Ccl5*, also called RANTES) were strongly decreased (*p* < 0.05; Figure 1E). Thus, implantation sites from *IL15Δ/Δ* rats exhibit robust gene expression differences compared to WT rats. Reduced NK cell number is likely to drive these differences.

### 3.2 Absence of PRF-containing cells in IL15Δ/Δ rat implantation sites

**FIGURE 2:**
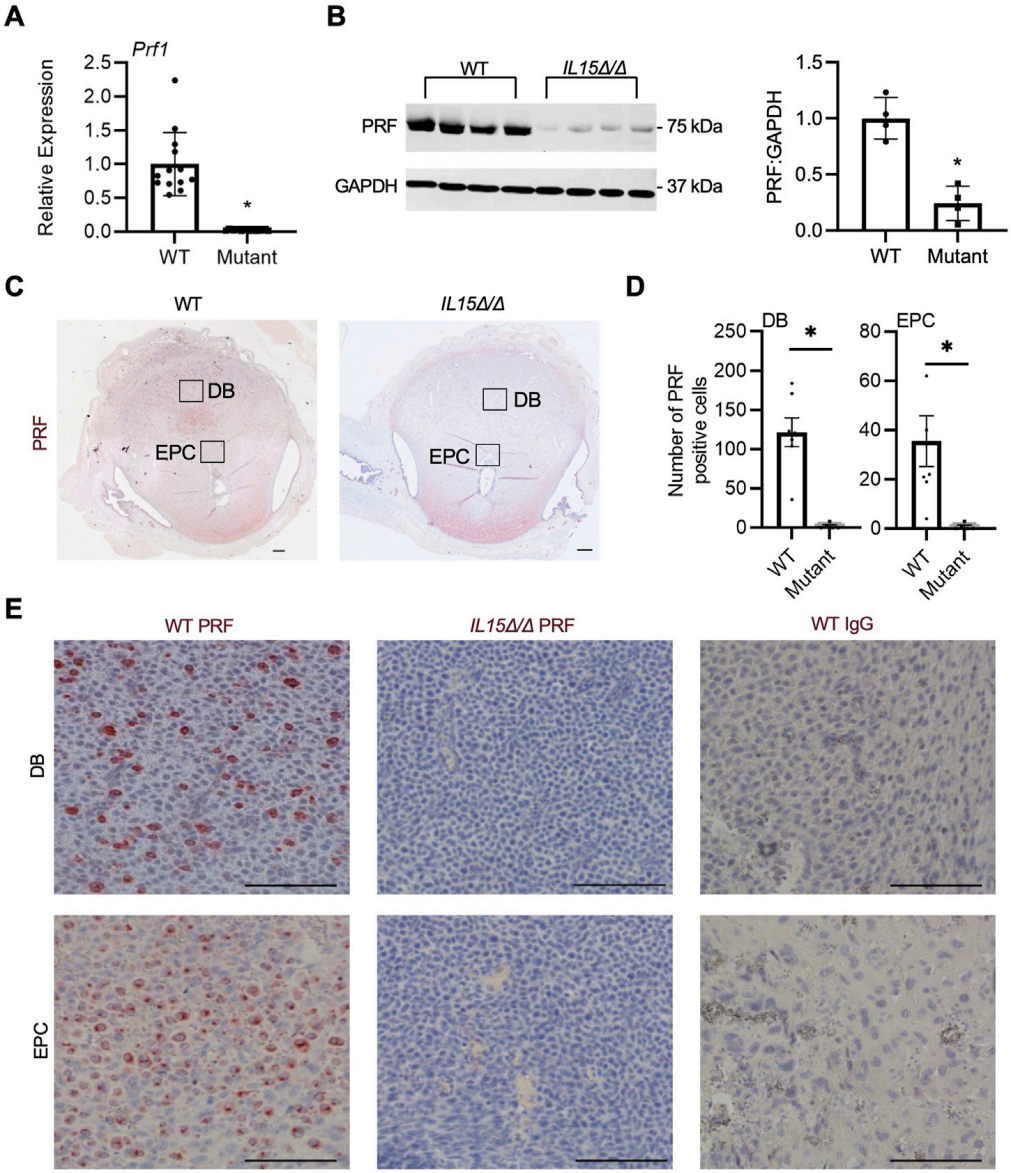
*IL15Δ/Δ* rats are devoid of PRF-containing cells in the uterus on GD 9.5. **(A)** Quantitative RT-PCR comparing *Prf1* levels between implantation sites from WT and *IL15Δ/Δ* (mutant) rats (*N* ≥ 13 implantation sites from at least 5 dams per group). **(B)** Western blot showing decreased PRF levels in implantation sites from *IL15Δ/Δ* rats compared to implantation sites from WT rats. GAPDH was used as a loading control. The histogram on the right shows results from densitometric analysis comparing PRF levels relative to GAPDH (*N* = 4 per group). **(C)** Images of PRF immunohistochemistry in WT *versus IL15Δ/Δ* implantation sites. Scale bar, 250 µm. The boxes show the approximate location of high magnification images in **(E)** for the decidua basalis (DB) and near the ectoplacental cone (EPC). **(D)** Quantification of PRF-positive cells in WT and *IL15Δ/Δ* implantation sites (*N* ≥ 5 implantation sites from different dams per group) in the DB and near the EPC. **(E)** Representative high magnification images showing PRF localization in the DB and near the EPC on GD 9.5. Please note the lack of PRF detection in *IL15Δ/Δ* decidua. The right panels show images captured following immunohistochemistry of WT implantation sites using a non-specific rabbit IgG in place of PRF. Scale bar, 50 µm. Panel **(A)** was analyzed using a linear mixed model followed by Tukey’s *post hoc* test; other experiments were analyzed using Student’s *t*-tests. Results represent means ± SEM. Data significantly different from controls (*p* < 0.05) are indicated by an asterisk (*).

Since IL-15 is required for NK cell development, our next goal was to confirm the absence of uterine NK cells in *IL15Δ/Δ* rat implantation sites. First, qRT-PCR was conducted on GD 9.5 implantation sites collected from WT and *IL15Δ/Δ* dams to detect *Prf1*, the gene encoding the pore-forming cytolytic protein PRF, which is highly expressed in NK cells and cytotoxic T lymphocytes. Since cytotoxic T lymphocytes are typically rare in the decidua (Erlebacher, 2013; Yang F. et al., 2019), we used *Prf1* expression as a proxy for NK cells. Not surprisingly, *Prf1* transcript levels were substantially downregulated (44-fold, *p* < 0.05) in implantation sites from *IL15Δ/Δ* rats (Figure 2A), which is consistent with results from the Clariom S analysis. To determine whether PRF is altered at the protein level, we performed Western blotting, and observed an 86% decrease in PRF protein levels in *IL15Δ/Δ* implantation sites compared to WT (Figure 2B). To confirm the localization of PRF (and thus NK cells), we conducted immunohistochemistry for PRF on GD 9.5 WT and *IL15Δ/Δ* implantation sites. At GD 9.5, WT rats had notable punctate PRF staining throughout the decidua basalis, including contiguous with the ectoplacental cone (which ultimately forms the main portion of the placental junctional zone). Little to no staining was detectable in the decidua of *IL15Δ/Δ* rats (Figures 2C–E). When quantifying the number of PRF positive cells, there was a 98.5% decrease in the number of PRF positive cells in the decidua and 97.2% decrease in PRF positive cells adjacent to the ectoplacental cone in *IL15Δ/Δ* rats compared to WT rats (*p* < 0.05, Figure 2D). Collectively, these results demonstrate that *IL15Δ/Δ* rats have greatly diminished numbers of PRF-containing uterine NK cells in the decidua including near the developing placenta.

### 3.3 Decreased OPN in the decidua of IL15Δ/Δ rats

**FIGURE 3:**
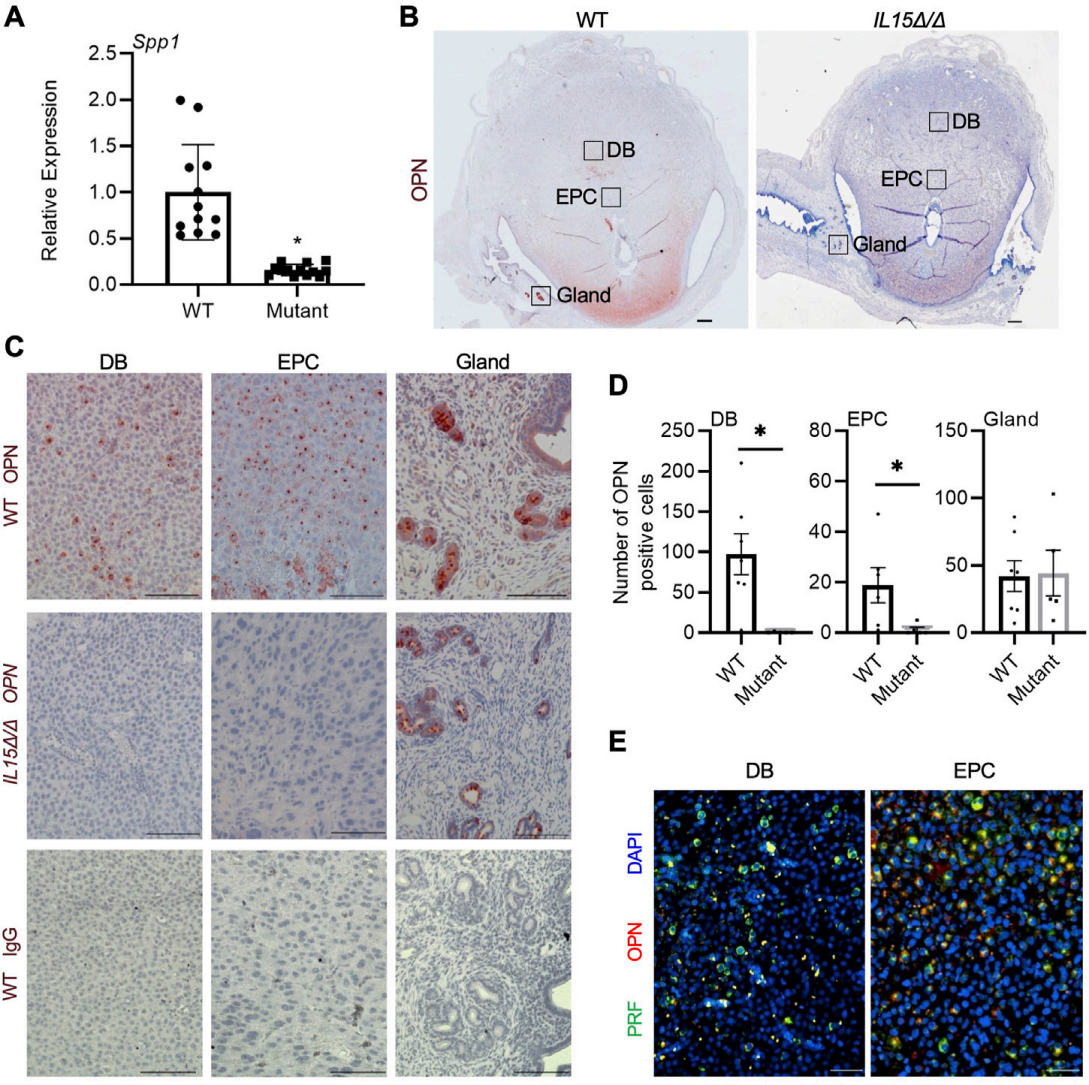
Reduced OPN in the decidua of *IL15Δ/Δ* rats. **(A)** Relative expression of *Spp1* mRNA in WT *versus IL15Δ/Δ* (mutant) implantation sites on GD 9.5 (*N* ≥ 13 implantation sites from at least 5 dams per group). **(B)** Images of OPN immunohistochemistry in WT *versus IL15Δ/Δ* implantation sites. Scale bar, 250 µm. The boxes show the approximate location of high magnification images in **(C)** for the decidua basalis (DB), near the ectoplacental cone (EPC), and at the periphery with uterine glands. **(C)** Higher magnification images showing OPN localization in the DB, near the EPC, and uterine glands of WT and *IL15Δ/Δ* implantation sites on GD 9.5. The bottom panels show images captured following immunohistochemistry of WT implantation sites using a non-specific mouse IgG_1_ in place of OPN. Scale bar, 50 µm. **(D)** Quantification of OPN-positive cells in DB, EPC, and uterine glands from WT and *IL15Δ/Δ* implantation sites (*N* ≥ 5 implantation sites from different dams per group). **(E)** Immunohistochemistry for PRF (green) and OPN (red) in a representative WT GD 9.5 implantation site. Images were taken of the DB and near the EPC. Please note that PRF and OPN frequently colocalize. Scale bars represent 50 μm. Panel **(A)** was analyzed using a linear mixed model followed by Tukey’s *post hoc* test; other experiments were analyzed using Student’s *t*-tests. Results represent means ± SEM. Data significantly different from controls (*p* < 0.05) are indicated by an asterisk (*).

OPN is a multifunctional protein that has previously been implicated in implantation, decidualization, and placentation as well as the development and homeostasis of NK cells (Erikson et al., 2009; Kang et al., 2014; Leavenworth et al., 2015; Pan et al., 2022). As *Spp1*, the gene encoding OPN, was one of the most significantly downregulated genes in the Clariom S array, we further investigated the expression and localization of OPN in rat implantation sites. Relative mRNA levels of *Spp1* were decreased by 87% in GD 9.5 *IL15Δ/Δ* implantation sites compared to WT (Figure 3A, *p* < 0.05). WT implantation sites had pronounced OPN staining in the decidua basalis including near the ectoplacental cone, in addition to robust staining in the uterine glands at the periphery of the implantation site. Comparatively, little to no OPN staining was detectable in implantation sites from *IL15Δ/Δ* rats near the ectoplacental cone (95% decrease, *p* < 0.05) or other regions of the decidua basalis (99.5% decrease, *p* < 0.05), although levels of OPN in uterine glands were comparable to WT rats (Figures 3B–D). To determine if OPN colocalizes with PRF, we co-stained for PRF and OPN in GD 9.5 WT implantation sites and found significant, albeit not complete, overlap (Figure 3E). Thus, our results strongly suggest that the main cellular source of OPN in the midgestation rat decidua is uterine NK cells.

### 3.4 Isolated uterine NK cells express OPN

**FIGURE 4:**
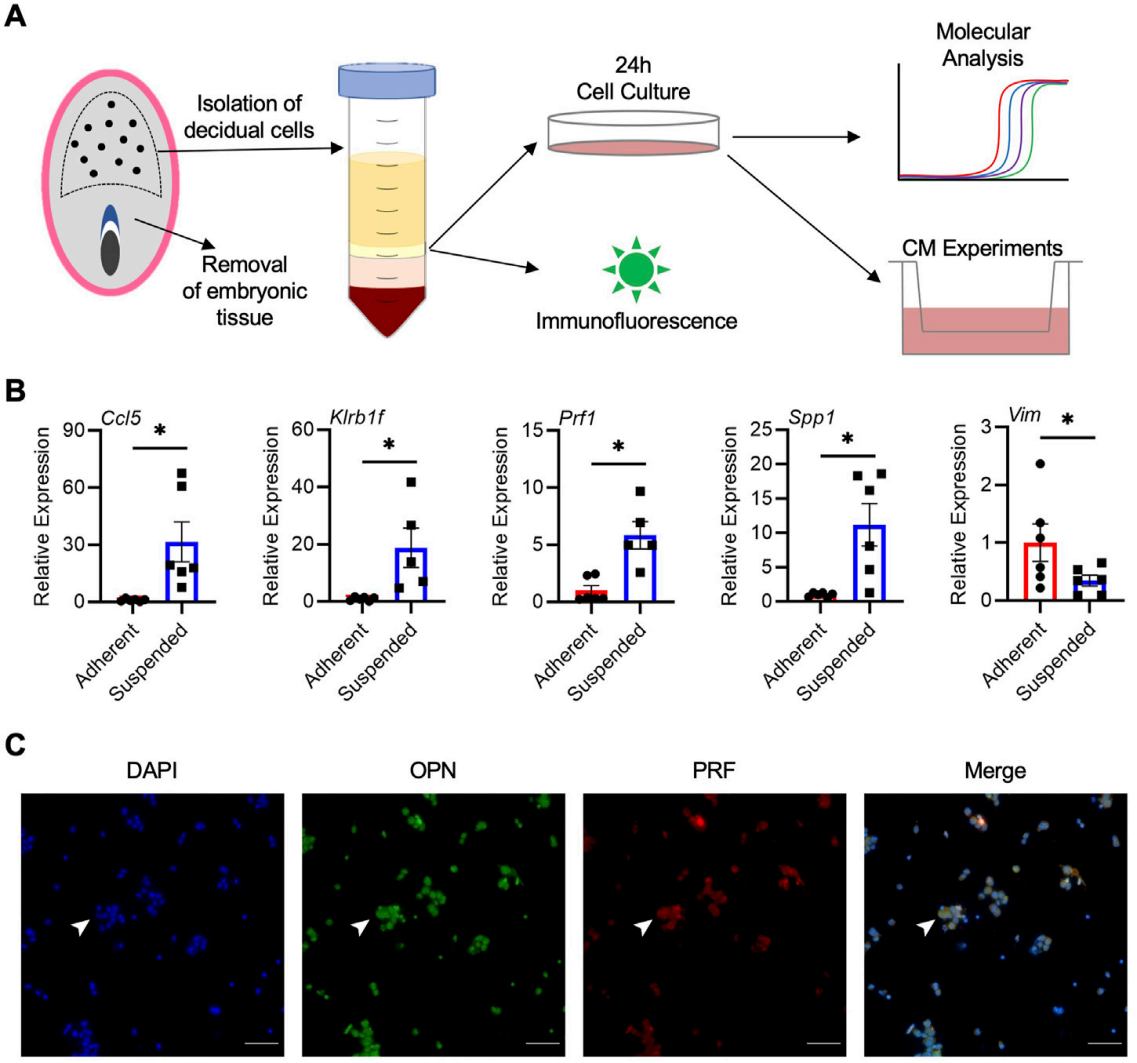
Isolation and enrichment of *Spp1*-expressing NK cells from the rat decidua. **(A)** Schematic depiction of experimental protocol. **(B)** Relative gene expression of *Ccl5*, *Klrb1f*, *Prf1*, *Spp1*, and *Vim* in adherent and suspended cells isolated from GD 9.5 decidua and cocultured for 24 h. Experiments were performed 6 times using different dams. **(C)** Non-adherent cells isolated from GD 9.5 decidua express both PRF and OPN. Arrowheads indicate a representative cell positive for both PRF and OPN staining. Scale bars, 50 μm. Results represent means ± SEM. Data significantly different from controls (*p* < 0.05; Student’s *t*-test) are indicated by an asterisk (*).

Our next goal was to determine whether cells from the rat decidua, including uterine NK cells, could be isolated and cultured. Following digestion of decidual tissue and density gradient centrifugation, isolated cells were placed in culture for 24 h (Figure 4A). Within 2 h, many cells adhered to the tissue culture plate, whereas others remained in suspension for the duration of culture. Compared to adherent cells, cells in suspension consistently had higher expression of NK cell markers *Ccl5* (31.5-fold), *Prf1* (5.8-fold), and *Klrb1f* (18.8-fold), as well as *Spp1* (11.2-fold; all *p* < 0.05; Figure 4B), indicating that NK cells are enriched within the suspended population. Adherent cells, on the other hand, exhibited morphologies characteristic of decidual stromal cells and had increased expression of *Vim*, the gene encoding vimentin (*p* < 0.05; Figure 4B). When cultures were extended past 24 h or when non-adherent cells were removed and cultured independent of adherent cells, cell viability in the suspended population was reduced, indicating that the adherent cells may produce factors important for viability of non-adherent cells. Conditioned media were collected from cocultures, and the presence of OPN was confirmed by ELISA, with an average value of 711 pg/mL based on decidual cell preparations from 4 dams. Further, immunofluorescence conducted on non-adherent cells showed that a large number of these cells stained positively for PRF and OPN, and were likely uterine NK cells (Figure 4C). Although most OPN and PRF staining appeared cytoplasmic, some nuclear localization of OPN was possible which may have been due to the processing of suspended cells for immunofluorescence or fluorescence microscopy. Collectively, results from these experiments indicate that a population of OPN-expressing uterine NK cells can be successfully enriched from rat decidua and maintained in culture.

### 3.5 Neutralizing OPN reduces rat trophoblast invasion toward decidual conditioned media *in vitro*

**FIGURE 5:**
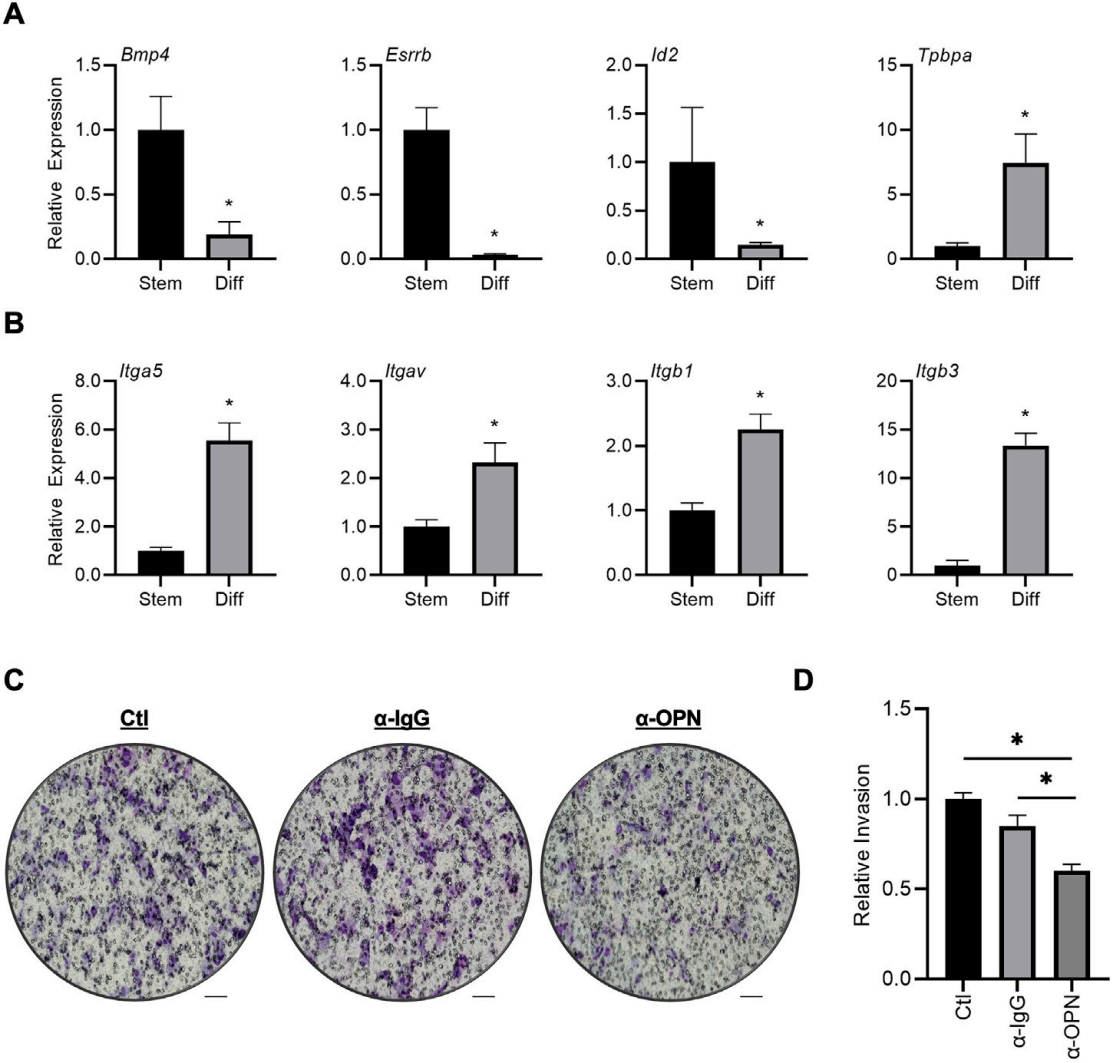
Neutralizing OPN in decidual conditioned media decreases invasiveness of rat trophoblast cells. **(A)** Expression of genes associated with the stem state (*Bmp4*, *Id2*, and *Esrrb*) or junctional zone trophoblasts (*Tpbpa)* after culturing TS cells in stem conditions (Stem) or after 6 days of differentiation (Diff). **(B)** Expression of genes encoding various integrin subunits (putative OPN receptors) in TS cells cultured in Stem or Diff conditions. **(C)** Representative images of Transwell membranes showing differentiated trophoblasts that invaded to the underside of the membrane after 24 h exposure to decidual conditioned media (Ctl), or conditioned media supplemented with non-specific IgG (α-IgG) or an OPN neutralizing antibody (α-OPN). Scale bars, 100 μm. The relative number of cells that invaded in each condition is graphically depicted in **(D)**. The experiment was conducted 4 times, with 3 membranes counted in each experiment. Results represent means ± SEM. Panel **(D)** was analyzed using a one-way analysis of variance followed by Tukey’s *post hoc* test; other experiments were analyzed using Student’s *t*-tests. Data significantly different from controls (*p* < 0.05) are indicated by an asterisk (*).

To determine the effect of OPN on trophoblast cell invasion, Matrigel-based invasion assays were performed using rat TS cells differentiated for 6 days. First, to confirm successful differentiation of rat TS cells, expression of several genes associated with stem and differentiated states was analyzed. After 6 days of differentiation, there was a substantial decrease in expression of genes associated with the stem state, including *Bmp4* (81%), *Esrrb* (97%), and *Id2* (86%), and increased expression of the junctional zone marker *Tpbpa* (7.4-fold, all *p* < 0.05, Figure 5A). Interestingly, although there was no change in *Cd44* expression during TS cell differentiation, genes encoding subunits of RGD-binding integrins exhibited increased expression in differentiated cells, including *Itga5* (5.5-fold), *Itgav* (2.3-fold), *Itgb1* (2.3-fold)*,* and *Itgb3* (13.4-fold) compared to TS cells maintained in stem conditions (Figure 5B, *p* < 0.05). Therefore, integrins expressed by differentiated trophoblasts may interact with OPN. To determine the effect of OPN on trophoblast invasion, rat TS cells were differentiated for 6 days, placed in Matrigel-coated Transwells, and then Transwells were placed in wells containing decidual conditioned media, or conditioned media supplemented with a non-specific control IgG antibody (α-IgG) or a neutralizing OPN antibody (α-OPN). Compared to the non-specific IgG condition, approximately 40% less cells invaded through the Matrigel following exposure to the OPN neutralizing antibody (Figures 5C, D, *p* < 0.05). Since blocking OPN in decidual conditioned media reduced trophoblast invasion *in vitro*, decreased levels of OPN are unlikely to underlie the precocious trophoblast invasion in *IL15Δ/Δ* rats.

## 4 Discussion

Uterine NK cells are thought to play key roles in the regulation of decidual development, placentation, and maternal allorecognition of the fetus (Croy et al., 2002; Acar et al., 2011; Faas and de Vos, 2017). To date, most studies investigating the *in vivo* function of uterine NK cells have used mouse models (Barber and Pollard, 2003; Bany et al., 2012; Fu et al., 2017). The use of mice has provided indispensable insight into the potential functions of uterine NK cells. However, trophoblast invasion in mice is inherently shallow (Ain et al., 2003). The importance of uterine NK cells in species that exhibit deep placentation and extensive trophoblast invasion is therefore not well understood. Since IL-15 is required for the development and survival of NK cells, a rat model of IL-15 deficiency was generated to discern the regulatory role of IL-15 (and consequently NK cells) on trophoblast invasion (Renaud et al., 2017). These *IL15Δ/Δ* rats exhibit robust, precocious trophoblast invasion by GD 13.5, suggesting that NK cells diminish or delay the onset of trophoblast incursion into the decidua. In the current study we performed gene expression profiling to identify genes that are differentially-expressed in the implantation site between WT and *IL15Δ/Δ* rats. We identified reduced expression of many genes associated with NK cell function in *IL15Δ/Δ* rat implantation sites, consistent with the notion that uterine NK cell numbers are diminished in *IL15Δ/Δ* rats. We also found altered levels of factors that may exert regulatory roles on trophoblast function through paracrine actions, such as OPN. These findings have important significance for pregnancy complications associated with dysregulated trophoblast invasion.

Through gene expression profiling, there were 257 genes that had significantly changed expression by at least 2-fold between WT and *IL15Δ/Δ* implantation sites. Genes that were upregulated in *IL15Δ/Δ* rats compared to WT rats were unexpectedly linked to neurological and sensory pathways. Since gene expression profiling was performed on the entire implantation site (including embryonic tissue), upregulated genes may reflect changes in early nervous system development. However, since the decidua is the major component of the implantation site at this stage of pregnancy, it is also possible that upregulated genes have additional functions in the decidua and pregnancy maintenance unrelated to their roles in neurological development. For example, the gene *Crym*, which is a member of the crystallin family studied extensively in the context of central nervous system development, lens formation, auditory function, and thyroid hormone regulation (Slingsby and Wistow, 2014), was upregulated in *IL15Δ/Δ* implantation sites. Crystallins have been identified in mouse and rat deciduas and protect decidual cells from oxidative and inflammatory stress-induced cell death (Iwaki et al., 1990; Zuo et al., 2014). Also upregulated in *IL15Δ/Δ* implantation sites was *Gria4*, a gene that encodes glutamate receptor 4 (GRIA4). While GRIA4 has primarily been implicated in neurotransmission and cognitive function, a role for GRIA4 has been described in preimplantation embryo development and in the context of pregnancy pathologies (Yeung et al., 2016; Špirková et al., 2022). Genes that were significantly downregulated in *IL15Δ/Δ* rat implantation sites compared to WT included *Prf1*, *Ccl5*, *Eomes*, *Gzmb* and *Il2rb*, which have all been implicated in NK cell development and function (Kim et al., 2011; Gordon et al., 2012; Fernandez et al., 2019), supporting the notion that diminished uterine NK cell number is a major feature of *IL15Δ/Δ* rat implantation sites.

PRF is a pore-forming cytolytic protein found in both T cells and NK cells (Kägi et al., 1994). Since the decidua lacks a prominent cytotoxic T cell population, we utilized PRF as a marker of uterine NK cells. *Prf1*/PRF was consistently decreased at both the transcript and protein level in *IL15Δ/Δ* implantation sites, corroborating that *IL15Δ/Δ* rats are devoid of uterine NK cells (Renaud et al., 2017). Similarly, studies that have disrupted either IL-15 or its receptor in mice also report reduced PRF in decidua (Bany et al., 2012). We further demonstrated that PRF-positive cells are prominent in WT rats in both the decidua basalis as well as adjacent to the ectoplacental cone, and that PRF-positive cells are reduced at both sites in *IL15Δ/Δ* rats. Notably, there appeared to be altered morphology or size discrepancies of cells in the WT and *IL15Δ/Δ* implantation sites. We conjecture that this may be due to the lack of uterine NK cells leading to altered decidual structure or cellular composition (Croy et al., 1997). Since the ectoplacental cone will ultimately give rise to the placental junctional zone (where invasive trophoblasts arise), it is enticing to speculate that uterine NK cells may directly influence junctional zone formation during early placental development. This may be achieved through direct receptor-ligand interactions as has been suggested in humans and mice (Gaynor and Colucci, 2017), as well as paracrine signaling networks and alterations to the decidual environment like matrix deposition, angiogenesis, and oxygen delivery. Further studies are warranted to uncover how uterine NK cells modify this aspect of placentation.

One of the most robustly downregulated genes in *IL15Δ/Δ* rat implantation sites was *Spp1*, the gene encoding OPN. OPN is a prime candidate as a potential regulator of placental development, due in part to its known role as a regulator of cell invasion (Qi et al., 2014; Wu et al., 2015) and its altered expression patterns in pregnancy complications (Xia et al., 2009; Özer et al., 2018; Long et al., 2019). OPN was robustly detected within the decidua basalis and adjacent to the ectoplacental cone, areas in which PRF staining was evident. Moreover, staining for OPN frequently colocalized with PRF, highly suggesting that NK cells are the main cellular source of OPN in the rat decidua. Consistent with this finding, OPN strongly localizes to uterine NK cells in mice (Nomura et al., 1988; Herington and Bany, 2007; Kramer et al., 2021). OPN staining was not readily detectable in the decidua of *IL15Δ/Δ* rats, which is similar to findings observed in the decidua of mice lacking IL-15 (Herington and Bany, 2007). Of note, OPN was also readily detectable in uterine glands at the periphery of the implantation site, where levels appeared unchanged between WT and *IL15Δ/Δ* rats. Previous research has identified OPN in uterine glands in multiple species including humans, non-human primates, rodents, sheep, pigs, and goats, indicating that the production of OPN by uterine glands is conserved among many mammals and is regulated by sex hormones (Ashworth and Bazer, 1989; Brown et al., 1992; Fazleabas et al., 1997; Garlow et al., 2002; Girotti and Zingg, 2003; Johnson et al., 2003; Joyce et al., 2005). Nevertheless, our results indicate that there are at least two sources of OPN production (glandular and uterine NK cell-derived) in the mid-gestation rat implantation site.

OPN is a matricellular phosphoprotein that is produced and secreted into the extracellular space to modulate cell function through interacting with molecules such as matrix proteins, CD44, and RGD-binding integrins including αvβ3 and α5β1 (Icer and Gezmen-Karadag, 2018). OPN-integrin interactions mediate implantation, blastocyst attachment, trophoblast proliferation, trophoblast invasion, and fetal growth in various species (Wu et al., 2015; von Wolff et al., 2004; Johnson et al., 2014; Qi et al., 2014; Zhou et al., 2016; Wang et al., 2018; Fu et al., 2017). Using a rat TS cell model, we showed that transcripts encoding the OPN-binding integrin subunits αv, α5, β1, and β3 were all increased when TS cells were differentiated for 6 days, and that neutralizing OPN from media conditioned by cells isolated from the decidua decreased trophoblast invasion. Therefore, OPN appears to exert a pro-invasive effect on *in vitro* differentiated rat trophoblasts. It should be noted that different forms of OPN have been described, including intracellular (not secreted) and nuclear (Kanayama et al., 2017; Icer and Gezmen-Karadag, 2018; Mahmud et al., 2020). Intracellular OPN has been described in peripheral NK cells with a critical role in IL-15 signaling as well as NK cell maturation and survival (Leavenworth et al., 2015). It is possible that the reduced levels of *Spp1* in *IL15Δ/Δ* rat implantation sites may be due, at least in part, to the lack of intracellular OPN. Further studies are warranted to determine whether intracellular OPN mediates IL-15 signaling in rat uterine NK cells.

We next isolated cells from the deciduas of WT animals. Isolation of uterine NK cells has been reported in human and mouse models. In rats, peripheral NK cells have been collected from late gestation placentas (Shields et al., 2018), but the enrichment of uterine NK cells has yet to be reported. Previous isolations of human uterine NK cells note expression of CD56, NKp46, and KIR2DL1, as well as granzymes A and B (Vacca et al., 2006). Here, we report that non-adherent cells isolated from the decidua are enriched in transcripts associated with NK cells including *Ccl5*, *Klrb1f*, and *Prf1*, whereas adherent cells expressed *Vim* and had morphologies consistent with decidual stromal cells. Higher expression of *Spp1* was detected in the non-adherent cells, providing further evidence that uterine NK cells are the main source of OPN in the GD 9.5 rat decidua. We cannot confirm at this point if other cell-types are present along with NK cells in the non-adherent population. Viability of non-adherent cells was best when cocultured with adherent cells, suggesting that NK cells (and potentially other non-adherent cells) may require signals from decidual stromal cells (Dunn et al., 2002; Yang H. L. et al., 2019). NK cells isolated from human decidua show increased expression of various cytokines and growth factors (i.e., *Vegfc*, *Cxcl10*, *Cxcl11*) when cocultured with trophoblasts or endothelial cells, which may influence their activation and survival (Lash et al., 2011; Gong et al., 2014). In future experiments it would be interesting to further isolate constituents of the decidua and decipher cell-specific contributions to the regulation of trophoblast invasion.

OPN has been implicated in inflammatory environments, and while it exists as a component of the extracellular matrix, it also functions as a cytokine (Lund et al., 2009). In human decidual tissue, the number of uterine NK cells positively correlates with the levels of secreted OPN, indicating a relationship between uterine NK cells and OPN, and a possible role for OPN in recruiting uterine NK cells to the pregnant uterus (Qu et al., 2008). Notably, the same study also reported reduced OPN levels in cases of recurrent spontaneous abortion corresponding with decreased uterine NK cell numbers. Combined with our findings of OPN produced by both NK cells and glands, it is possible that OPN contributes to a positive feedback loop mediated through IL-15 signaling to facilitate recruitment and maintenance of uterine NK cells during pregnancy. Consistent with this possibility, in mice OPN promotes the expansion of common lymphoid progenitor cells from which NK cells arise (Kanayama et al., 2017). OPN may thus exert multiple functions at the maternal-fetal interface, including recruitment, maintenance, and survival of uterine NK cells as well as effects on junctional zone formation and placental development.

This study has several limitations. First, our study focused on GD 9.5 because NK cells are prevalent at this time and placentation is in its nascency. Additional experiments over the course of gestation would be informative. Second, while our study provides insights into gene expression differences between WT and *IL15Δ/Δ* implantation sites, and many of these differences appear to be related to the reduced number of uterine NK cells in *IL15Δ/Δ* rats, we were unable to delve further into distinct NK cell subsets or maturation status. Wider availability of antibodies targeting rat proteins and newer technologies such as single cell RNA sequencing or cellular indexing of transcriptomes and epitopes followed by sequencing may enable a more robust investigation of uterine NK cell subsets in rats. It is important to note that gene expression profiling was performed on whole GD 9.5 implantation sites (which included the developing embryo). The Clarion S gene expression array data is, therefore, expected to also include expression changes in the embryo, in addition to the placenta, decidua and uterine wall. Finally, our study selected one transcript that was differentially-expressed between WT and *IL15Δ/Δ* implantation sites, *Spp1*/OPN, for further investigation. We determined that OPN is produced by NK cells (and uterine glands), and appears to promote invasion of rat trophoblasts *in vitro*. However, *in vitro* experiments do not recapitulate the complex physiological environment at the utero-placental interface, and it is possible that NK cell-derived OPN exerts a more complex function *in utero*, such as altering trophoblast proliferation and differentiation patterns. Indeed, the absence of NK cells in *IL15Δ/Δ* rats is associated with enhanced junctional zone development and precocious trophoblast invasion, which is paradoxical to OPN’s pro-invasive effect on rat trophoblasts *in vitro*. Additional NK cell-derived products may contribute to the regulation of trophoblast differentiation and invasion, and the summative effect of NK cells on trophoblast functions may be the convergence of multiple signals.

In summary, we have profiled gene expression changes in the rat implantation site between WT and *IL15Δ/Δ* rats. We further showed that OPN is produced by uterine NK cells, and we speculate that it may play a role in controlling processes related to placentation and decidualization. Our discoveries provide new insights into the roles of IL-15 in pregnancy, and by extension provide a detailed characterization of uterine NK cells in rats. Given the advantages of the rat as an experimental model of hemochorial placentation exhibiting deep trophoblast invasion, findings from this study advance our understanding of the complex cellular dynamics of the maternal-fetal interface.

## Data Availability

The datasets presented in this study can be found in online repositories. The names of the repository/repositories and accession number(s) can be found below: Gene Expression Omnibus accession number: GSE216395.
